# Bifunctional epoxy coatings containing new nano-hybrid based on lignin for decoration and gamma radiation shielding

**DOI:** 10.1038/s41598-026-53047-9

**Published:** 2026-05-30

**Authors:** Khlood S. Abdel Zaher, Walaa M. Abd El-Gawad, Wageeh Ramadan, Galal A. M. Nawwar

**Affiliations:** 1 Green Chemistry Department, National Research Centre, 33 El-Bohouth St., Dokki, Giza, 12622 Egypt; 2 Polymers and Pigments Department, National Research Centre, 33 El-Bohouth St., Dokki, Giza, 12622 Egypt; 3 Radiation Protection and Safety Department, Hot Laboratories and Waste Management Center, Egyptian Atomic Energy Authority (EAEA), Cairo, Egypt

**Keywords:** Epoxy coatings, Rice straw, Lignin, Color, Gamma radiation shielding, Chemistry, Environmental sciences, Materials science, Nanoscience and technology

## Abstract

Recently, researchers focused on utilizing agricultural waste, such as rice straw, to prepare eco-friendly green compounds that have industrial applications, rather than burning it, which leads to environmental problems. In this study, a new Mn (lignin/silica/fatty acid) nano-hybrid Mn(LSF) was deposited from the black liquor of rice straw and then mixed with epoxy to prepare bifunctional epoxy coatings for decoration and gamma radiation shielding. The chemical structure and morphology of the Mn(LSF) nano-hybrid were confirmed by FTIR, XRF SEM, EDX, mapping, and TEM. After the synthesis, Mn(LSF) nano-hybrid was integrated into epoxy resin with three proportions (e.g., 2%, 4%, and 8%), and then each coating was painted on plastic substrates with three thicknesses (e.g., 100 μm, 150 μm, and 200 μm) to investigate the effect of the concentration and thickness on the degree of color and gamma radiation shielding. The results revealed that the evaluated Mn(LSF) nano-hybrid is synthesized in nanoscale and its main elements, such as manganese, silica, and carbon, are distributed homogenously in the lignin base. Moreover, the color measurements elucidated that the lightness decreases with increasing the concentration and the thickness; therefore, the darkness increases. Furthermore, the γ-ray shielding ability of the prepared coatings containing Mn(LSF) nano-hybrid throughout an energy range of 0.662, 1.173, and 1.332 MeV emitted by the radioactive sources Cs-137 and Co-60 was measured using the narrow beam transmission method and a NaI (Tl) scintillation detector. The measurements depict an enhancement in the linear attenuation coefficient with increasing the Mn(LSF) nano-hybrid substitution in the coatings. The linear attenuation coefficient increased by 17.83%, 18.30%, and 18.14% at 0.662 MeV, 1.173 MeV, and 1.332 MeV, respectively, as Mn(LSF) nano-hybrid rise across the concentration of 2% to 8%.

## Introduction

 Open burning of agricultural residues leads to serious environmental problems, including soil and water pollution and the depletion of essential soil nutrients. During combustion, valuable elements such as carbon, nitrogen, and sulfur are released into the atmosphere in the form of harmful gases such as methane, nitrogen oxides, and ammonia. These emissions intensify air pollution, contribute to ozone formation, and increase the concentration of fine particulate matter, which poses severe risks to public health, particularly respiratory and cardiovascular diseases^1,2^.

In recent years the utilization of natural or renewable plant biomass, such as rice straw for the development of functional polymer materials has attracted significant interest due to ecological, economic, and environmental considerations^3^.

Rice straw (RS) is mainly composed of lignin, silica, cellulose, phenolic and fatty acids. Among these components, lignin is one of the most abundant macromolecules on the Earth and consists of three different phenylpropane units: p-hydroxyphenyl (H), syringyl (S) and guaiacyl (G). Its phenolic hydroxyl structure allows incorporation into various polymer systems. Lignin has been widely utilized in polymer blends and composites, including phenolic resin, polyurethane foams, polyethylene, polypropylene, polystyrene, poly (vinyl alcohol), epoxy resins. It also acts as an antioxidant, stabilizing oxygen free radicals^4–8^.

Lignin has shown promise for gamma-ray shielding when combined with metal–organic composites, as it contributes to materials with strong attenuation capacity. Owing to its resistance to radiation damage, lignin serves as a functional component in such composites, enabling their use in applications like transporting radioactive materials^9,10^.

Silica is another component of rice straw and plays an important role in composite materials. Due to the presence of silanol (Si–OH) group, silica exhibits hydrophilic behavior because of its good compatibility with epoxy through chemical and hydrogen bonding interactions^11,12^.

In earlier work, lignin–silica hybrids were applied to polypropylene, where incorporating silica into lignin through hybridization enhanced the thermal stability of PP/silica–lignin composites^13^.

Although Pure silica (SiO₂) exhibits limited gamma-ray shielding due to its low density, its combination with heavy metal significantly improves the effective atomic number and density, making silica-based composites suitable for gamma radiation shielding application^14,15^.

The increased use of radioactive materials in several fields, such as nuclear power, industry, and medicine, needs the generation of environmental and effective radiation shielding materials to decrease the harm to both humans and the environment. Traditional shielding materials, such as lead, are effective due to their high density and atomic number, which help attenuate radiation.

However, these materials have significant disadvantages, such as toxicity, weight, and environmental issues with handling and disposal. As a result, scientists have concentrated on preparing lightweight and sustainable alternatives, can effectively shield against radiation^16–18^.

Epoxy-based coating is a promising alternative that may give good adhesion, mechanical stability, and simplicity of preparation. According to recent studies, shielding of gamma radiation may be improved by incorporation of radioactive elements with high atomic numbers, such as lead oxide and tungsten oxide; into epoxy coatings. These substances may increase the effective atomic number and density of coating, thereby enhancing their radiation attenuation characteristics^19^.

In recent research, rice straw black liquor was utilized to prepare (lignin / silica/fatty acid) Nano hybrid contains different metals (Zn, Cu, Ca, and Al) which showed multifunctional properties when added to polymer matrix. These hybrid materials showed improved chemical and physical properties for rubber and epoxy matrices. However, their use as a natural gamma -radiation shield, especially for epoxy matrices loaded with manganese, is not yet fully investigated^20–25^.

Recent studies have demonstrated significant progress in the development of advanced polymer-based gamma radiation shielding materials using both conventional and eco-friendly approaches. For instance, silicone rubber and polymer composites reinforced with metal oxides and industrial by-products, such as iron slag, TiO₂, SnO₂, Bi₂O₃, and CdO, have shown enhanced thermal stability and radiation attenuation performance, particularly when nano-sized fillers are incorporated^26–28^.

In addition, several studies have explored sustainable shielding materials derived from agricultural and waste resources, such as rice straw and natural binders, demonstrating promising radiation protection capabilities through both experimental and simulation approaches^29,30^. Furthermore, high-density fillers such as tungsten carbide have been successfully used to significantly improve attenuation efficiency in polymer matrices, although often at relatively high filler loadings^31^.

These studies highlight that the radiation shielding performance of polymer composites strongly depends on filler type, particle size, dispersion, and loading content, while also emphasizing the growing interest in developing lightweight, sustainable, and multifunctional shielding materials.

Previous studies have demonstrated the potential of manganese-based epoxy composites for radiation shielding; however, most reported systems rely on high inorganic filler loadings and lack sustainability^32^. Despite the promising properties of lignin/silica- based hybrids, their use as eco-friendly gamma radiation shields from agricultural waste particularly manganese containing lignin/silica/ fatty acid nano-hybrids in epoxy coatings remains limited, especially at low filler loadings.

Therefore, the main aim of this study was to prepare a new cost-effective Mn (lignin/silica/fatty acid) nano-hybrid from rice straw black liquor to be incorporated into epoxy resin with different concentrations to provide bifunctional coatings for both decoration purposes and gamma ray shielding.

## Materials and experimental techniques

### Materials

The black liquor used in this study was obtained from the alkaline solar pulping of rice straw, collected from El-sharkia government, Egypt. The rice had an average length of approximate length of 1.0 m. The pulping experiment of rice straw was carried out in closed polyethylene bags following the procedure described in^33^.

Manganese sulfate (MnSO_4_), sodium hydroxide, were analytical-grade products purchased from Sigma-Aldrich. Epoxy resin consists of bisphenol A and polyamide as the curing agent. This ratio was obtained from Kemapoxy, Egypt. Other chemicals and solvents used in this study were of laboratory reagent grade.

### Preparation of the Mn(LSF) nano-hybrid

One liter of rice straw pulping black liquor pH 12 was treated with 40 gm Manganese sulfate while stirring with a magnetic stirrer. The reaction was left overnight, and the obtained precipitate was filtered and washed with tap water, and then oven-dried at 105 °C to give the Mn(LSF) nano-hybrid. Upon addition of manganese sulfate to the alkaline black liquor, a decrease in pH was observed to pH7 indicating interactions between Mn²⁺ ions and the functional groups of lignin and silica.

### Preparation of the coatings

In this work, a ball mill was utilized to prepare new epoxy coatings containing Mn(LSF) nano-hybrid with three proportions of 2%, 4%, and 8%. Firstly, Mn-composite was dispersed in a mixed solution of xylene and N-butanol (weight ratio is 2:1) under ultrasonic dispersion for 0.5 h. Next, the dispersed solution was mixed with the epoxy resin in a ball mill for 2 h. Three formulations were prepared. After that, each coating was painted on plastic substrates with three thicknesses, 100 μm, 150 μm, and 200 μm, to determine the effect of the thickness on the degree of gamma radiation shielding by using a film applicator^25,34^. The coating thickness was measured using a digital coating thickness gauge. The measurements were carried out following the general principles of ASTM D7091 for nondestructive dry film thickness measurement of coatings. Multiple readings were taken at different locations on each sample to account for spatial variations, and the average value was reported as the film thickness. Additionally, the plastic sheet is polyethylene terephthalate (PET) with a thickness of 0.5 mm and a composition of (C_10_H_8_O_4_)_n_, and it is additive-free, optically transparent, commercial grade, and was obtained from Modern Plast Co. in Egypt. The formulations are shown in Table 1.

**Table 1:** Paint formulations.

Coatings with different thickness ( µ)	Ingredients (gm)	
	Epoxy	Mn(LSF) nano-hybrid
Group I (2%)		
2% 100 μm	98	2
2% 200 μm	98	2
Group II (4%)		
4% 100 μm	96	4
4% 200 μm	96	4
Group III (8%)		
8% 100 μm	92	8
8% 200 μm	92	8

### Characterization of the synthesized Mn-nanocomposite and coatings

Transmission electron microscopy (TEM) Particle Sizing Systems\ZPW388 obtained from Santa Barbara, Calif., USA, was used. Scanning electron microscopy (SEM)/energy-dispersive X-ray analysis (EDX) techniques using micro-analyzer electron probes (JEOL JX 1230 and JEOL JX 2840) Japan, respectively, were used to determine the shapes and sizes of the synthesized Mn-nanocomposite and coatings. FT-IR spectra of the Mn-nanocomposite and coatings were obtained with a JASCO FTIR-4100 E (Japan) operating in absorption mode in the wave number range of 4000–400 cm^− 1^ by the prepared compounds mixing with KBr (potassium bromide) discs. The spectra were obtained at a resolution of 4 cm^− 1^ using a model ATR PRO450-S single reflection measuring attachment. XRF Elemental Analysis by Wavelength Dispersive X-Ray Fluorescence Spectrometry using Axios advanced, Sequential WD_XRF Spectrometer, PANalytical 2005.

The X-ray diffraction (XRD) data were measured by the modern diffractometer Bruker d8 advance, Germany, using copper source Kα radiation (λ = 1.5406 Å) at 40 mA, 40 kV, in the 2θ range 5°-80°, step size 0.05° using automatic divergence slit and scan rate of 0.6°/sec.

### Methods of testing and evaluation of coatings

#### Color measurements

Color measurements were carried out according to previously reported CIELab-based procedures using a Lovibond Tintometer RT 100 color tintometer^25^. The color of the coatings was spectrally analyzed via the CIELab method, where the L* value represents color lightness on a scale from 0 to 100, with 100 indicating a perfect reflecting diffuser and 0 corresponding to black or very dark colors. The a* and b* coordinates represent the chromatic axes positioned orthogonally and intersecting at the center. According to this system, a color cannot simultaneously be red and green or blue and yellow. Positive a* values indicate red, while negative values indicate green. Positive b* values correspond to yellow, whereas negative values correspond to blue^25^.

#### Gamma radiation shielding measurements

Gamma radiation shielding measurements were conducted following a standard experimental setup reported in earlier studies^35–37^. A sodium iodide NaI(Tl) scintillation detector, housed in a 16 mm thick lead shield with a 5 mm diameter collimator and coupled to a computerized multichannel analyzer, was employed to determine the γ-ray attenuation coefficients of the prepared coatings containing Mn(LSF) nano-hybrid. By a narrow beam transmission geometry produced by the gamma sources Co-60 and Cs-137, the attenuation parameters were measured for slabs were arranged successively way and placed with minimum interstitial space to avoid the negative effect of the air gaps. Sodium iodide (NaI) crystals are widely used scintillation materials in γ-ray spectrometry due to their high light output and effective compatibility with photomultiplier tubes. Gamma-ray spectra of specimens were measured using a sodium iodide Na (Tl) scintillation detector (CANBERRA, model: 802) connected to a computerized analyzer. The analyses of gamma spectra were achieved using Genie 2000 (version 3.1) software^38,39^.

Radioactive sources with an activity of 5 µCi, including Cs-137 (0.662 MeV) and Co-60 (1.173 and 1.332 MeV), were utilized. No additional gamma peaks or measurable changes in activity were observed following irradiation with Cobalt-60 and Cesium-137, indicating that no isotopic transformation occurred. Moreover, the energies of the emitted gamma rays are well below the threshold required to induce photonuclear reactions, confirming the absence of any nuclear processes. Photonuclear reactions typically require gamma-ray energies on the order of several MeV, commonly around or above 8 MeV, depending on the target nucleus^40,41^.

**Fig. 1:**
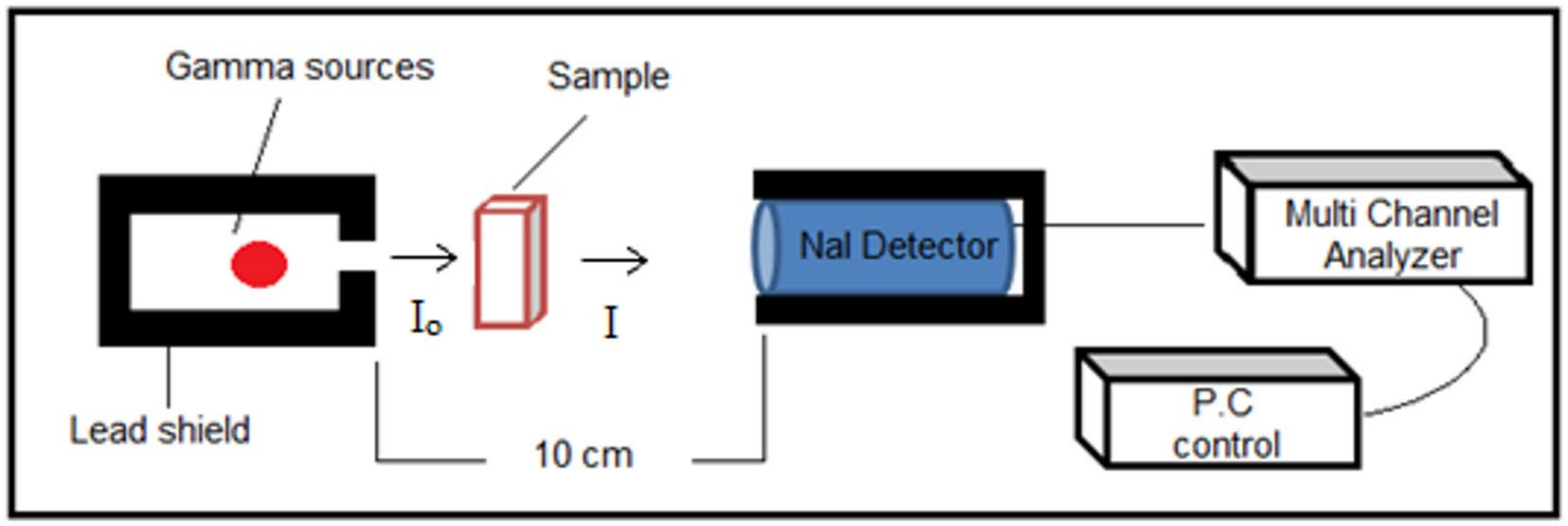
Schematic measuring diagram of the ^137^Cs and ^60^Co gamma sources with the prepared shield material.

The experimental setup used for γ-radiation measurements is illustrated on Fig. 1. The linear attenuation coefficient (µ) was calculated by comparing the transmitted radiation intensity through the sample (Iₓ) of thickness (x) with the incident intensity (I₀), using the Beer-Lambert law^35^.

1 $$\:\mathrm{I}_\mathrm{x}=\left[\mathrm{I}_\mathrm{o}{\mathrm{e}}^{-{\upmu\:}\mathrm{x}}\right]$$

Key γ-ray shielding parameters, including the half-value layer (HVL), tenth-value layer (TVL), and mean free path (MFP), were calculated following established relations^42^. HVL and TVL represent the absorber thickness required to reduce the incident γ-ray intensity to one-half and one-tenth of its initial value, respectively, while MFP denotes the average distance between successive photon interactions. These parameters were calculated using Eqs. (2–4):

2 $$\:\mathrm{H}\mathrm{V}\mathrm{L}=\left[\raisebox{1ex}{$\mathrm{ln}2$}\!\left/\:\!\raisebox{-1ex}{${\upmu\:}$}\right.\right]$$

3 $$\:\mathrm{T}\mathrm{V}\mathrm{L}=\left[\raisebox{1ex}{$\mathrm{ln}10$}\!\left/\:\!\raisebox{-1ex}{${\upmu\:}$}\right.\right]$$

4 $$\:\mathrm{M}\mathrm{F}\mathrm{P}=\left[\raisebox{1ex}{$1$}\!\left/\:\!\raisebox{-1ex}{${\upmu\:}$}\right.\right]$$

The experimental linear attenuation coefficient (µ) was calculated from the measured intensities and specimen thickness, and the experimental results were finally compared with both theoretical values obtained using XCOM (https://physics.nist.gov/PhysRefData/Xcom/html/xcom1.html).

The relative error $\:E\left(\%\right)$ between theoretical and experimental results was calculated to assess agreement using Eq. 5.

5 $$\:E\left(\%\right)\:=\:\left(\frac{{\mu\:}_{theo}-{\mu\:}_{exp}}{{\mu\:}_{exp}}\right)\times\:100$$

Effective electron density (N_eff_) and effective atomic number (Z_eff_) are essential for determining the photon transmission rate, especially in mixtures. One of the most important metrics for evaluating the effectiveness of shielding materials is the Z_eff_. It cannot be precisely expressed by a single number since it depends on the photons’ energy for each composite. For compounds, the effective atomic number can be calculated as follows^43,44^:

6 $$\:{Z}_{eff}=\frac{\sum\:_{i}{f}_{i}{A}_{i}{\left(\frac{\mu\:}{\rho\:}\right)}_{i}}{\sum\:_{j}\frac{{A}_{j}}{{Z}_{j}}{\left(\frac{\mu\:}{\rho\:}\right)}_{j}}$$

7 $$\:{N}_{eff}=\frac{{N}_{A}{n}_{t}{Z}_{eff}}{{\sum\:}_{i}{n}_{i}{A}_{i}}$$

In the shielding material, A_i_ and Z_i_ are the atomic weight and atomic number of the Mn-nanocomposite element, respectively, and f_i_ is the ratio of the Mn-nanocomposite element number to the total element number in the material. The Z_eff_ has a high correlation with the N_eff_. The formula for a compound is

8 $$\:{N}_{eff}=\frac{{\left(\frac{\mu\:}{\rho\:}\right)}_{c}\:}{{\sigma\:}_{e}}\:\:=\:\frac{{N}_{A}\:}{M}\:\:{Z}_{eff}\sum\:_{i}{n}_{i}$$

where σ_e_ is the electronic cross section provided by, n_i_ is the total number of atoms, and M is the particle’s mass;

9 $$\:{\upsigma\:}\mathrm{e}\:=\:\frac{1\:}{n}\sum\:_{i}{n}_{i}\frac{{{\upsigma\:}}_{i}\:}{{\mathrm{Z}}_{i}}$$

## Results and discussion

### Nano-hybrid Characterization

The formation of the Mn(LSF) nano-hybrid was initially supported by the physicochemical changes observed during the synthesis process. Upon addition of manganese sulfate to the highly alkaline black liquor (pH ≈ 12), a noticeable decrease in pH was recorded, indicating interactions between Mn²⁺ ions and the deprotonated functional groups of lignin (–O⁻) and silica (Si–O⁻). These interactions promote the formation of coordination linkages (Mn–O–C and Mn–O–Si), suggesting that the resulting Mn(LSF) nano-hybrid is a chemically integrated system rather than a simple physical mixture. This structural integration is further confirmed by the subsequent FTIR, XRF, and XRD analyses.

#### (FT-IR) spectra and (TEM)

**Fig. 2:**
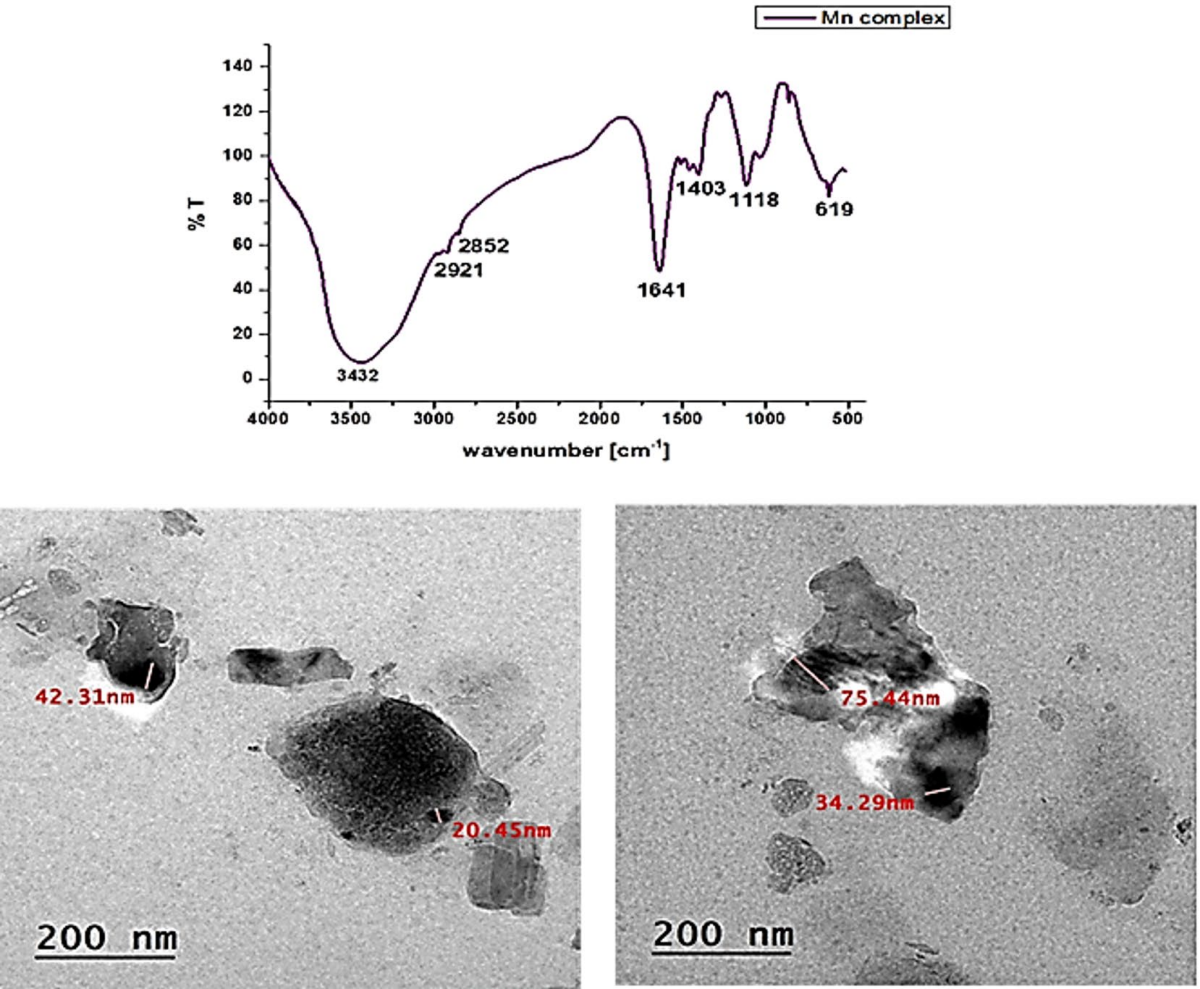
FTIR and TEM micrographs of Mn (LSF) nano-hybrid.

Fourier transform infrared (FT-IR) spectra for complexes Mn(LSF) nano-hybrid Fig. 2a showed that the absorption broadband appeared at 3432 cm^− 1^, is attributed to the presence of stretching vibrations of the alcoholic and phenolic OH groups of lignin, and the alkyl groups appeared at 2921, 2852 cm^− 1^. Additionally, the intense band that appeared at 1641 cm^− 1^ is characteristic of the stretching vibration of the carbonyl groups of fatty acids. Besides, the characteristic band at 1403 cm^− 1^ and 1118 cm^− 1^ indicate the presence of stretching vibrations of C-O bonds and aromatic rings.

Transmission electron microscope (TEM) is used to determine the size and shape of complex particles, as shown in Fig. 2. TEM of Mn(LSF) nano-hybrid in Fig. 2b shows that the silica particles have bright shape deposited on dark platelet lignin, the two shapes overlap each other, which ranges between 20 and 75 nm From the obtained data, it is clear that the particles of complexes appeared on Nano scales besides, lignin and silica congregated, merged together, and overlapped with each other.

#### X-ray fluorescence (XRF) analysis

The composition of the complex was determined through XRF analysis, as detailed in Table 2. The results indicate a notably high Loss on Ignition (LOI) value of 59.02%, which primarily suggests a significant presence of lignin and fatty acids. Also, XRF analysis reveals that manganese (Mn) accounts for approximately 27.27%, while silica is present at around 8.04%.

**Table 2:** X-ray fluorescence (XRF) analysis of the Mn (LSF) nano-hybrid.

Main constituents	%
SiO_2_	8.04
MnO	27.27
Fe_2_O_3_^tot^.	0.07
Na_2_O	1.18
NiO	0.054
SO_3_	1.61
Al_2_O_3_	0.17
CaO	0.10
ZnO	1.39
K_2_O	0.38
P_2_O_5_	0.07
Cl	0.09
LOI	59.02
MgO	0.51
SrO	0.005
Br	0.003
Co_3_O_4_	0.048

#### The X-ray diffraction (XRD)

**Fig. 3:**
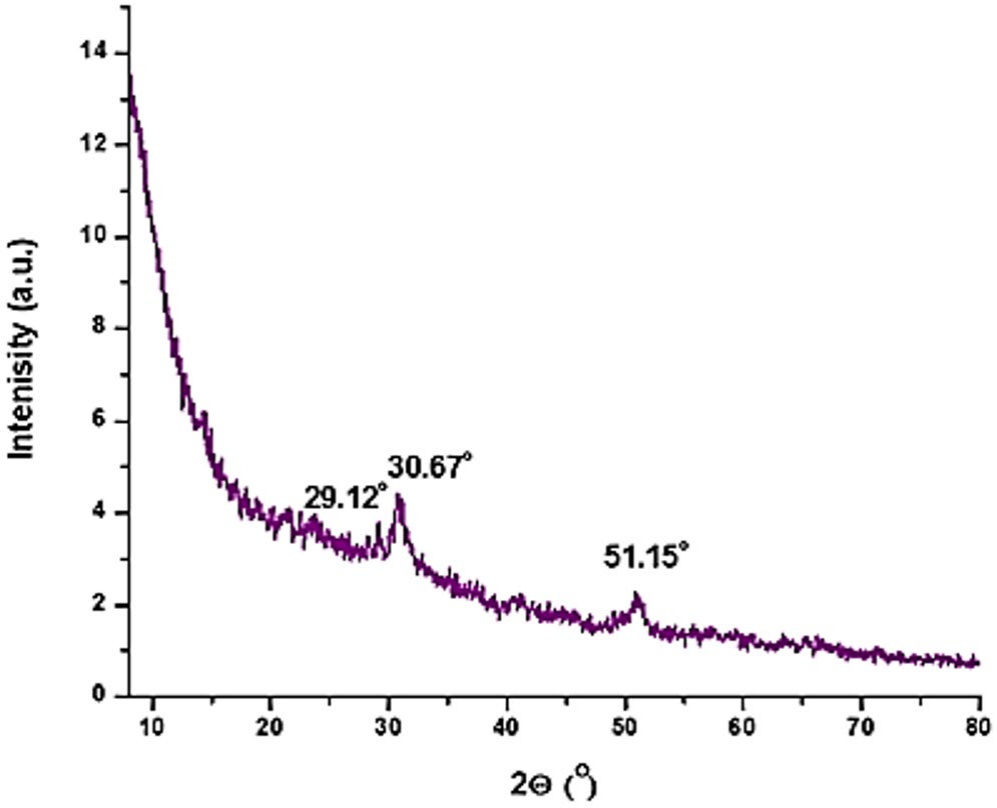
XRD of Mn(LSF) nano-hybrid.

The X-ray diffraction (XRD) pattern of the prepared Mn(LSF) nano hybrid (Fig. 3) indicates a predominantly amorphous structure, which is clear from the broad diffuse band around low diffraction angles. This behavior is characteristic of lignin- silica hybrid materials, where the organic lignin polymer and amorphous silica network don’t allow the formation of long-range crystalline order. A broad peaks low intensity diffraction pattern is also found around 29.12°, 30.67°, and 51.15° (2θ)^45,46^. In addition to amorphous halo. The low intensity diffraction indicates that these diffractions could be due to some short-range ordering around manganese silicate, these observations indicate that in the nano hybrid contains manganese and silicate. Such amorphous nature and homogenous distribution of manganese offer advantages in polymer fabrication and gamma radiation shield.

#### Scanning electron microscope (SEM)

The surface topography and composition of the hybrid fillers were analyzed using Scanning Electron Microscopy (SEM), a technique employing a focused electron beam to generate high-resolution images based on beam-sample interactions. SEM micrographs of the Mn(LSF) nano-hybrid fillers demonstrate a proper distribution of silica, Manganese, and clusters within the base lignin matrix.

**Fig. 4:**
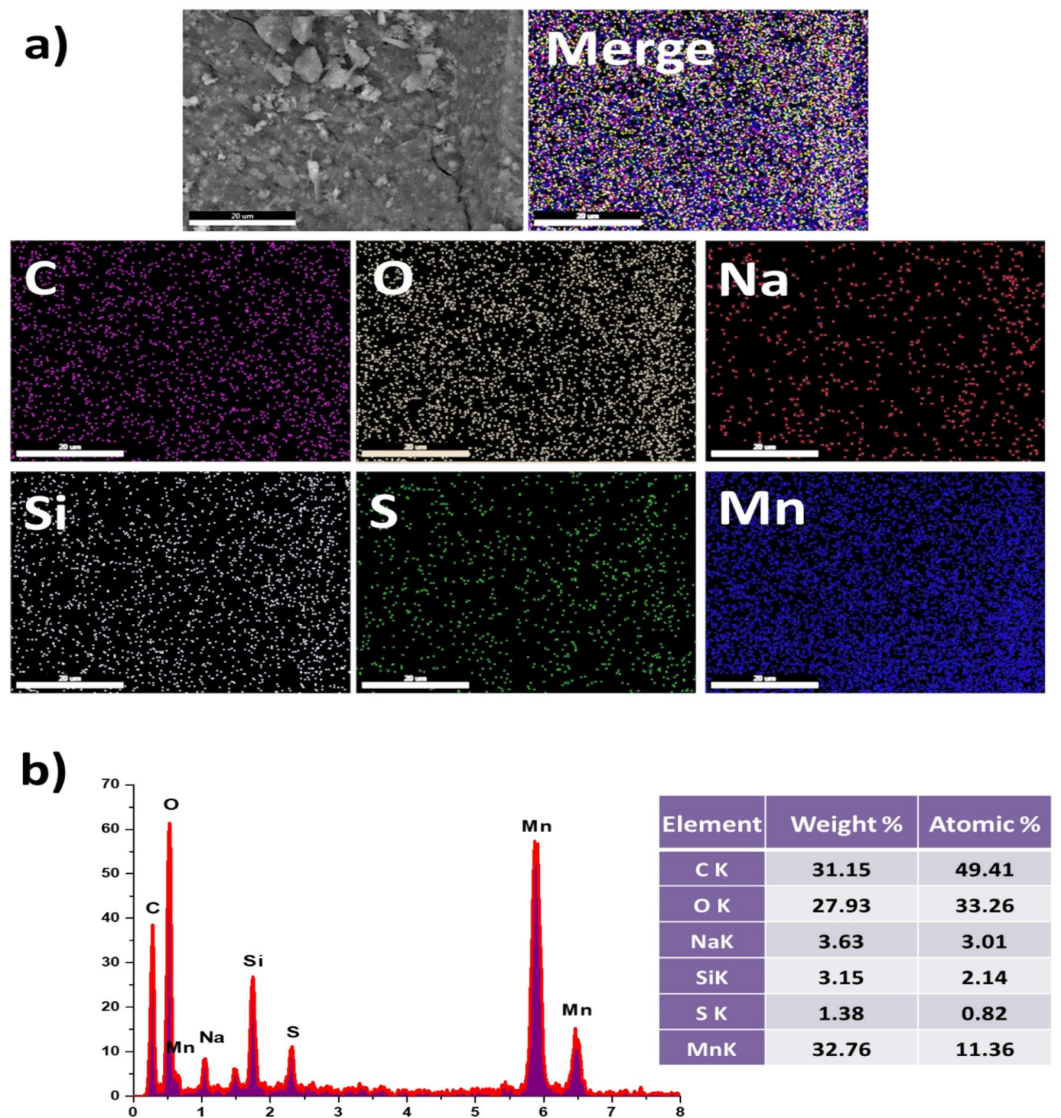
(**a**) SEM, mapping and (**b**) EDX of Mn(LSF) nano-hybrid.

The EDAX analysis (Fig. 4) showed that the organic content of the Mn(LSF) complex was C 31.15% wt. (representing the lignin and fatty acids), O 27.93% wt., Mn 32.76% wt., Si 3.15% wt, S 1.38%., and Na 3.63% wt.

Also, the EDAX mapping of the Mn(LSF) nano-hybrid (Fig. 4) shows a homogenous distribution of atoms, which indicates crosslinking between them^25^.

#### Zeta potential of Mn(LSF) nano-hybrid

**Fig. 5:**
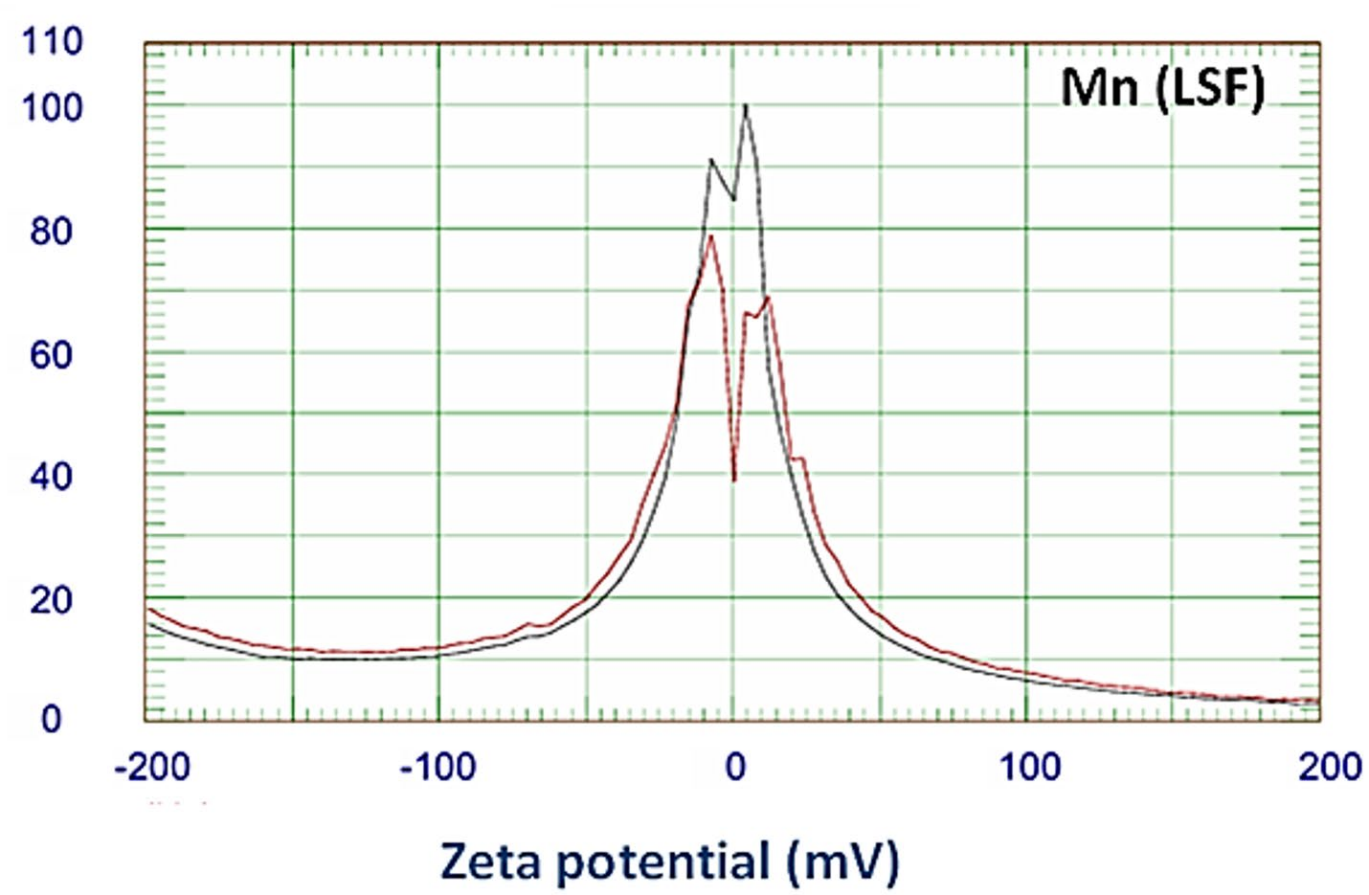
Zeta potential of the Mn(LSF) nano-hybrid.

Zeta potential is widely recognized for providing insight on the stability of nanoparticles in the media in which they are dispersed. The particle is considered stable if the potential is within the range of + 30 to − 30 mV. Figure 5 illustrates Zeta potential of the Mn(LSF) nano-hybrid. It falls within the indicated range, demonstrating that they are stable and do not aggregate under the intended usage conditions^25^.

### Morphological investigating of the coatings using SEM

**Fig. 6:**
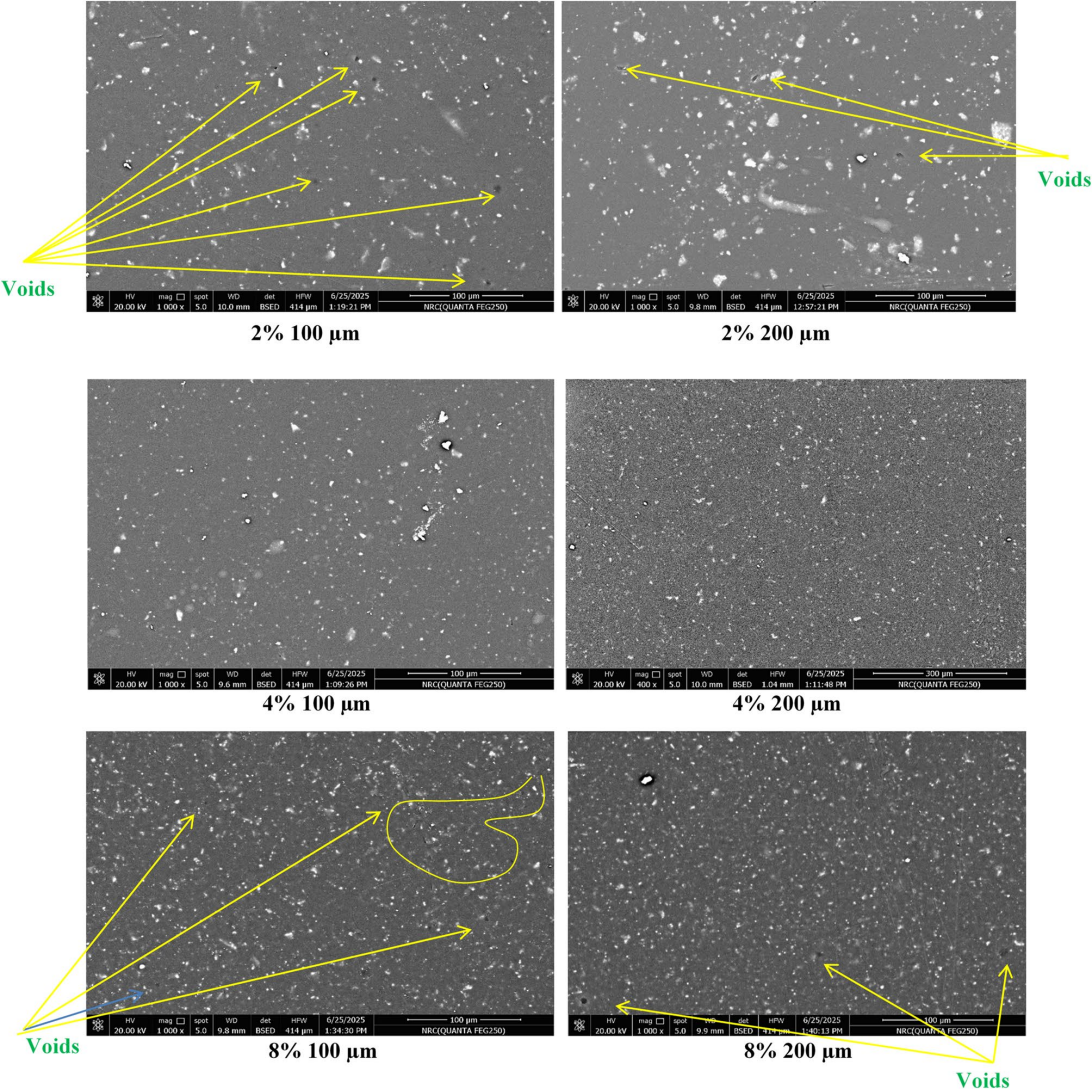
SEM photos of the prepared coatings.

The SEM images provide insights into the structure and dispersion of the Mn(LSF) nano-hybrid within the epoxy polymeric matrix Fig. 6, analyzing two thicknesses (100 μm and 200 μm) among three groups. In Groups I and II, the thicker films demonstrate a denser packing of Mn-nanocomposite particles, which presumably contributes to a rougher surface. This improved texture could enhance features such as wear resistance and adhesion. In Group III, both thicknesses also present denser films. All films demonstrate a commendable distribution within the epoxy polymeric matrix. However, a lower number of voids is noted in the films of Groups I and III. The holes identified in Group I may originate from the lower concentration of Mn(LSF) nano-hybrid, where the particles did not attain complete coverage, leading to gap formation inside the polymeric matrix. In contrast, the high concentration of group III with high thickness could lead to a significant surface roughness and a highly compact texture, due to increased particle agglomeration and clustering. While this clustering can boost durability and toughness, it can result in deficiencies if the particles are not properly dispersed, thereby affecting the integrity of coatings.

Consequently, the coating containing 4% Mn(LSF) nano-hybrid with thickness 200 μm is anticipated to display the ultimate durability. This is related to both the increased particle density and thickness, resulting in a homogeneous layer with little cavities or cracks and significant distribution^47,48^.

### EDX and mapping analysis of 4% 200 μm

**Fig. 7:**
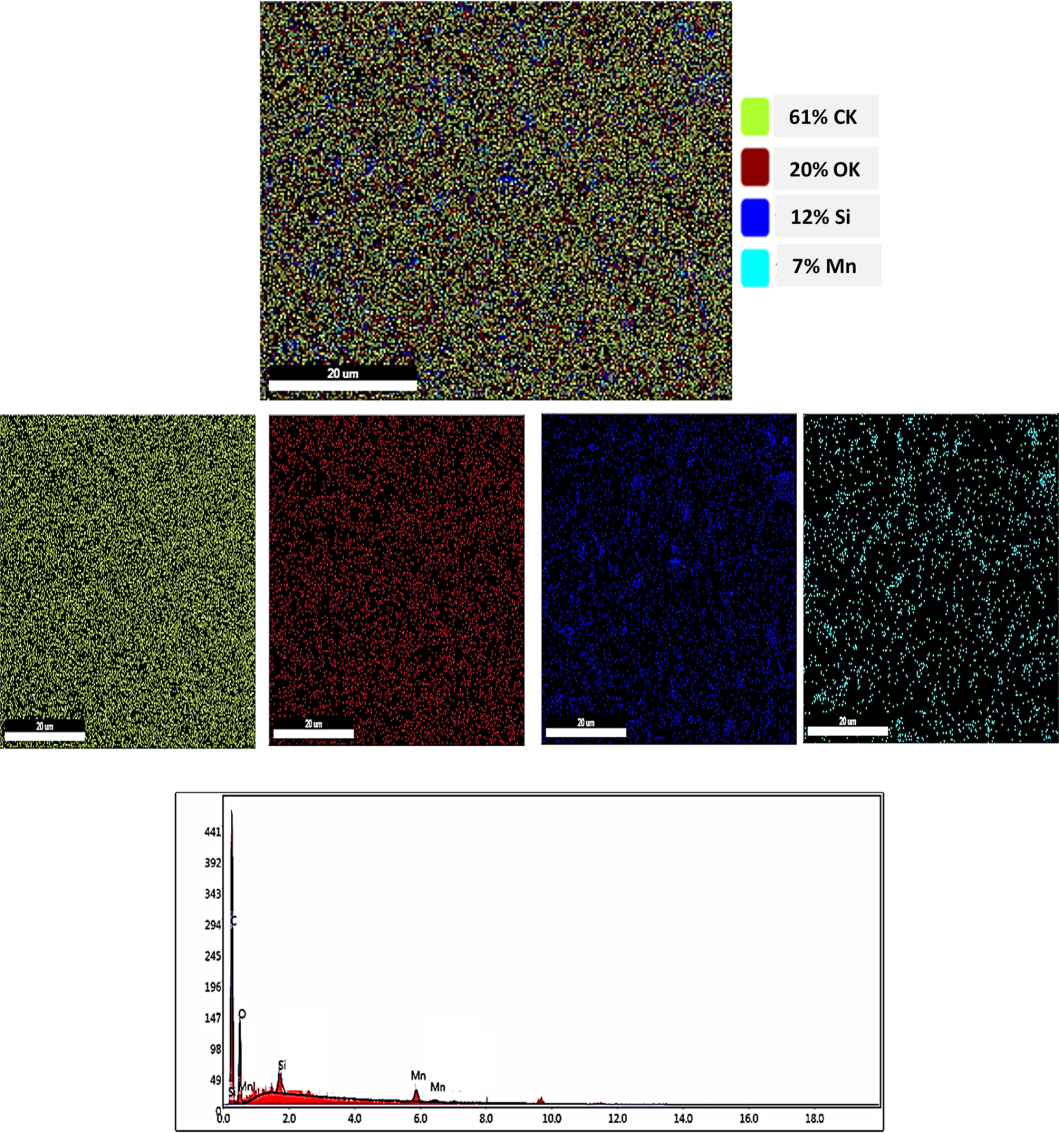
EDX and mapping analysis of coating has 4% 200 μm.

To further study the distribution and composition of this beneficial 4% 200 μm coating within the polymeric matrix, mapping and EDX analysis were performed. Figure 7 show the mapping and EDX analysis of the optimum coating which is 4% 200 μm. This technique is essential for investigating the microstructural characteristics of the coatings by detecting the elemental composition and distribution and of materials within the polymeric coatings. Herein, the mapping images show the good distribution of all elements of Mn(LSF) nano-hybrid (e.g., Mn, C, O, Si) in the epoxy matrix. Moreover, both EDX and mapping analysis declare that the main element is carbon followed by O because they form the main composition of epoxy, besides they exist in Mn(LSF) nano-hybrid. Additionally, Si and Mn elements appeared in the images which prove the integration of the composite in the polymeric film.

### Color investigations

The color of coatings is one of their most specific features. Therefore, figuring out their color coordinates is essential. Table 3 presents the results for the color coordinates of the prepared coatings. The results reveal that an increase in coating thickness and concentration from 100 µ to 200 µ contributes to a noticeable decrease in lightness (L*). This shows that thicker coatings can have lower reflection or absorb more light, resulting in darker hues. This property may be essential for applications where the appearance and dark color of the coating is critical, such as in automobile or decorative coatings. Moreover, in group I, the b* values increase from 100 µ to 200 µ which reflects a significant increase in yellow tones^25^. In contrast, in groups II and III, raising thickness promotes increasing in the a* values with richer hue, which may improve visual attractiveness in consumer products. The low lightness may be appropriate for applications requiring camouflage or stealth, such as industrial conditions or military. While the coatings have low Mn(LSF) nano-hybrid concentrations and thickness, they provide fairly brilliant colors with warm tones. These coatings may be appropriate for decorative applications when vibrancy is needed^49^.

**Table 3:** Color measurements of coating.

Coatings with both thickness	L*	a*	b*
Group I (2%)			
100 μm	59.87	13.73	23.74
200 μm	46.95	17.61	26.09
Group II (4%)			
100 μm	55.51	14.21	30.39
200 μm	40.41	17.53	21.06
Group III (8%)			
100 μm	32.16	11.29	7.77
200 μm	26.54	13.83	6.79

### Gamma radiation shielding

**Fig. 8:**
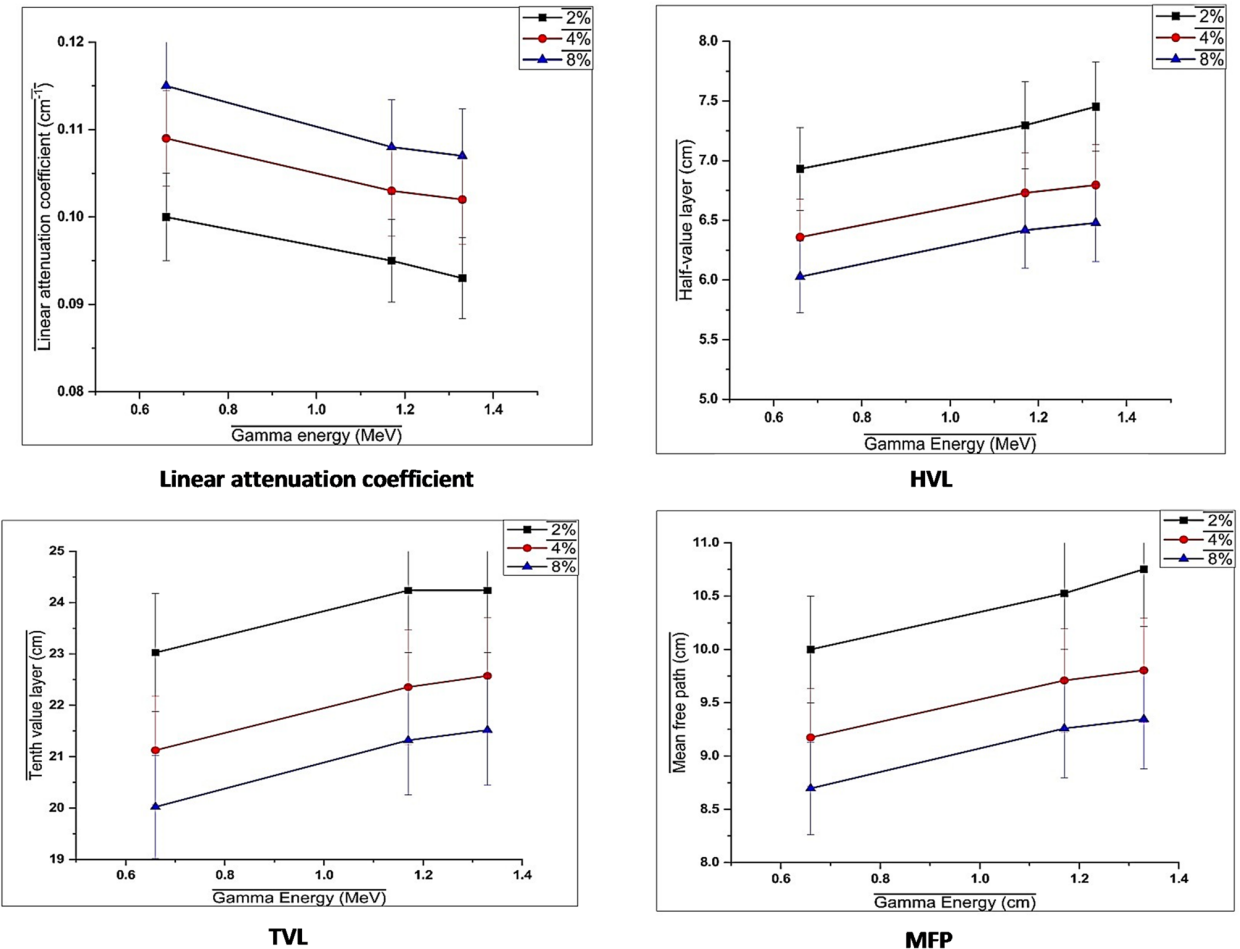
Radiation shielding results of coatings irradiated with Cs137 and Co60 gamma rays sources.

The radiation shielding findings of the coatings containing Mn(LSF) nano-hybrid clearly demonstrates the combined effect of gamma-ray energy and Mn concentration on photon attenuation. The change in behavior from photoelectric absorption at reduced energies to Compton scattering at higher levels, where the possibility of interaction is lower, is indicated by the linear attenuation coefficient (µ) decreasing for all coatings as gamma energy increases as shown in Fig. 8.

There are three different ways for photons to interact with matter depending on their energy. The photoelectric effect, Compton scattering, and pair production are all phenomena that occur at different energy levels: low, medium, and high, respectively. Where the probability of a Compton reaction occurring is proportional to Z and photon energy (E) according to Z/E. It is well known that the µ values indicate how well the shield materials are able to deflect harmful gamma rays which is dependent on photon energy and material density^50,51^.

As a result, the mean free path (MFP), half-value layer (HVL), and tenth-value layer (TVL) all increase with energy, demonstrating that thicker coating layers are needed to attain the same degree of attenuation toward higher-energy photons^52^.

On the other hand, higher µ values and lower HVL, TVL, and MFP illustrate that raising the Mn(LSF) nano-hybrid content from 2 to 8% enhances the shielding at all energy levels. This enhancement may be due to the high atomic number and mass density of Mn, which encourage stronger photon-matter interactions and reduce the distance that gamma photons travel before interaction^53^.

The formation of a complex of Mn within the lignin-silica-fatty acid matrix also helps to create a more homogeneous and compact structure, which increases attenuation efficiency. The coating containing 8% of Mn(LSF) nano-hybrid is the most effective shielding among the prepared coatings, especially at lower gamma energies from Cs-137. However, efficiency gradually decreases at higher Co-60 energies, which is consistent with the inherent drawbacks of slightly low-Z coatings^54^.

Table 4 compares the theoretical and experimental linear attenuation coefficients (LACs) of the reference concrete with concretes containing different percentages of activated carbon across a wide gamma-ray energy range. The 8% composite have the highest density, generally exhibits the highest theoretical linear attenuation coefficient values. Notably, the mixtures show competitive and, in some cases, enhanced experimental linear attenuation coefficient (µ) values with acceptable theoretical-experimental deviations with difference don’t exceed 7% .

**Table 4:** Theoretical and experimental linear attenuation coefficients and relative deviations for mixtures at different gamma-ray energies.

Energy (MeV)	Blank			2%			4%			8%		
	XCOM	Exp	E%	XCOM	Exp	E%	XCOM	Exp	E%	XCOM	Exp	E%
**0.66**	0.098	0.092	6.52	0.102	0.099	3.03	0.111	0.109	1.83	0.121	0.115	5.22
**1.17**	0.088	0.086	2.33	0.094	0.095	− 1.05	0.108	0.103	4.85	0.115	0.108	6.48
**1.33**	0.087	0.084	3.57	0.091	0.093	− 2.15	0.107	0.102	4.90	0.112	0.107	4.67

**Fig. 9:**
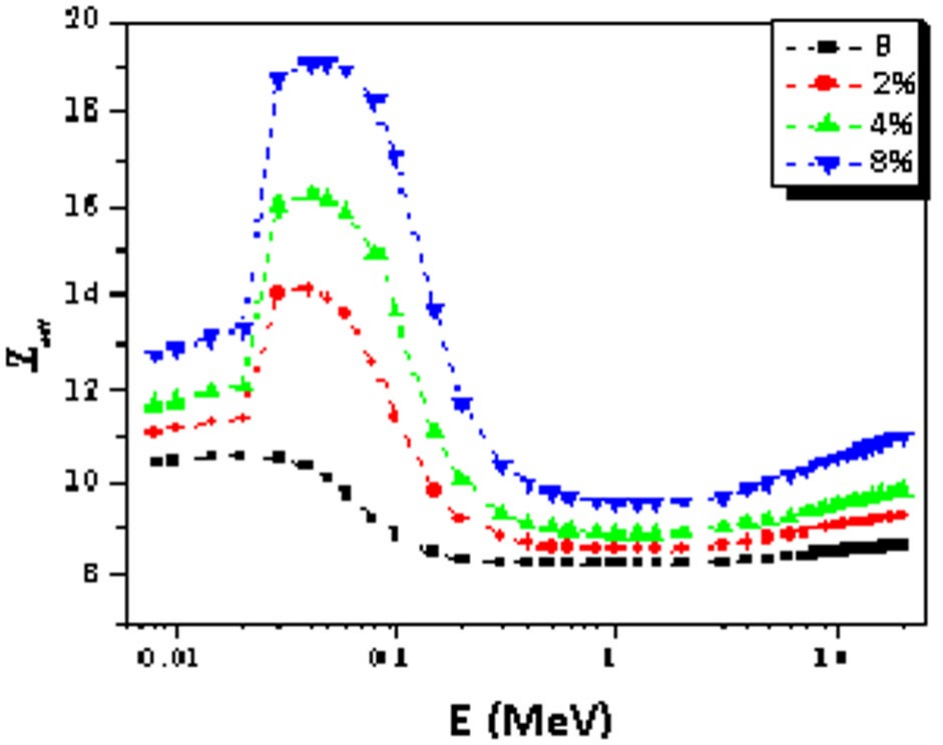
The Z_eff_ gamma energy’s distribution for the studied composites.

Distinct peaks in the effective atomic number (Z_eff_) are observed. The maximum Z_eff_ values for B, 2%, 4%, and 8% composites are 10.61, 14.13, 16.25, and 19.14 at photon energies of 0.02, 0.03, 0.04, and 0.05 MeV, respectively, as shown in Fig. 9. At higher photon energies (E ≥ 4 MeV), pair production (PP), which strongly depends on the atomic number (Z), becomes the dominant interaction mechanism. Consequently, Z_eff_ increases again with energy, particularly for materials with higher atomic numbers. Among all the investigated glass composites, the 8% sample exhibits the highest Z_eff_ values, making it the most suitable candidate for gamma-ray shielding applications.

**Fig. 10:**
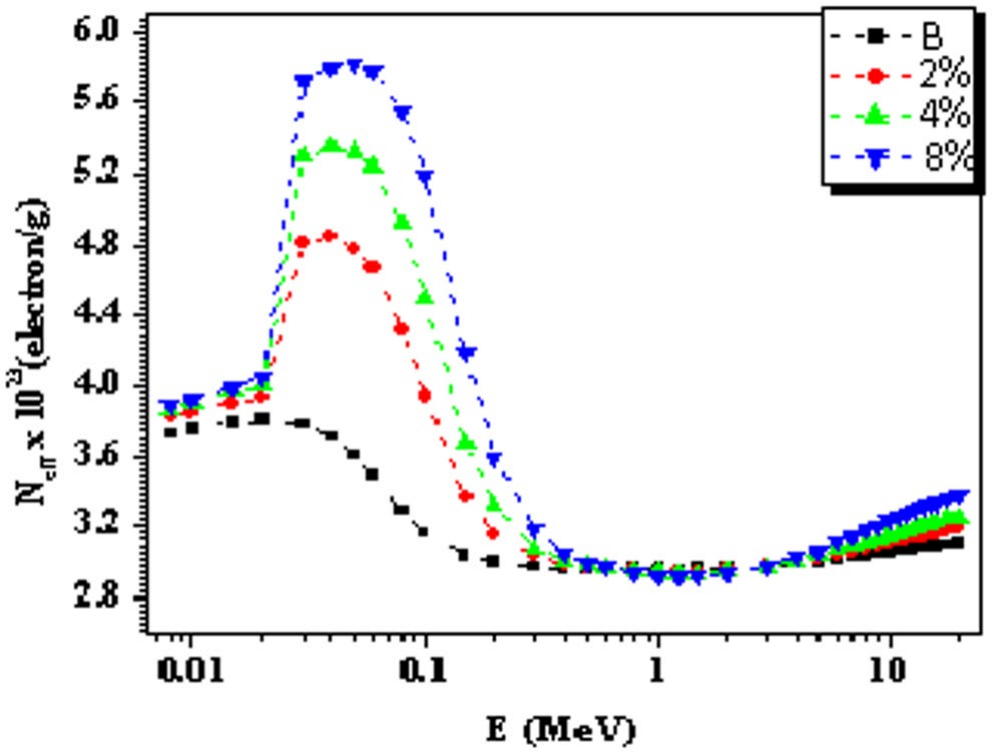
The N_eff_ gamma energy’s distribution for the studied composites.

Figure 10 illustrates the distribution of the effective electron density (N_eff_) as a function of gamma-ray energy for the studied composites. The behavior of N_eff_ is governed by the three primary mechanisms of gamma-ray interaction, each dominating within a specific energy range. In the low-energy region (Eγ < 0.5 MeV), Neff is relatively low because it depends on the binding energy of orbital electrons. In this range, gamma-ray interactions occur predominantly with tightly bound inner-shell electrons, meaning that only a limited number of electrons contribute effectively. In the intermediate energy region (0.5 < Eγ < 5 MeV), Neff reaches its maximum values and becomes approximately equal to the total electron density (ρe). This is because gamma-ray interactions are mainly governed by Compton scattering, which involves quasi-free (loosely bound) electrons. At high energies (Eγ > 5 MeV), corresponding to the pair production (PP) region, gamma rays interact primarily with the nuclear Coulomb field. As a result, N_eff_ becomes effectively negligible, since the interaction is no longer with atomic electrons but with the nucleus. At photon energies of 0.02, 0.03, 0.04, and 0.05 MeV, the maximum N_eff_ values for B, 2%, 4%, and 8% composites are 3.91 × 10^23^, 4.82 × 10^23^, 5.31 × 10^23^, and 5.83 × 10^23^ electrons/g, respectively.

## Conclusions

A new nano-hybrid of Mn(LSF) was successfully prepared from rice straw black liquor and then incorporated into epoxy to produce bifunctional coating for both decoration purposes and gamma radiation protection. The chemical composition of the Mn(LSF) hybrid was confirmed by FTIR and XRF, revealing a high lignin and fatty acids content (59.02%), along with manganese (27.27%) and silica (8.04%). Furthermore, morphological characterization using SEM, EDX, mapping, and TEM confirmed the nanoscale structure and uniform atomic distribution of the Mn(LSF) hybrid in the prepared coatings, which indicated good compatibility and dispersion of the filler.

The optical analysis revealed that the systematic reduction in lightness with the increasing amounts of the nano hybrid Mn(LSF) (2–8 wt%) and the thickness (100–200 μm was appropriate for decorative purpose. In parallel, gamma-ray shielding performance improved with increasing the amount of the nano hybrid Mn(LSF), which was evident from the increase in the linear attenuation coefficient by a relative 18% at energies of 0.662,1.173, and 1.332 MeV. This could be due to the influence of the high atomic number element, such as manganese, and the nano-hybrid structure.

Overall, this study demonstrates the effective valorization of rice straw waste into multifunctional nano-hybrid filler providing an eco-friendly way for developing epoxy coatings that combine aesthetic properties with efficient gamma radiation shielding.

## Data Availability

All data is provided within the manuscript.
